# SEOM clinical guidelines for the treatment of Hodgkin’s lymphoma

**DOI:** 10.1007/s12094-015-1429-1

**Published:** 2015-10-26

**Authors:** A. Rueda Domínguez, J. Alfaro Lizaso, L. de la Cruz Merino, J. Gumá i Padró, C. Quero Blanco, J. Gómez Codina, M. Llanos Muñoz, N. Martinez Banaclocha, D. Rodriguez Abreu, M. Provencio Pulla

**Affiliations:** Área de Oncología y Hematología, Hospital Costa del Sol, Autovía A-7, km 187, 29603 Marbella, Málaga Spain; Servicio de Oncología Médica, Instituto Oncológico de Guipúzcoa, San Sebastian, Spain; Servicio de Oncología Médica, Complejo Hospitalario Regional Virgen Macarena, Seville, Spain; Servicio de Oncología Médica, Hospital Universitari de Sant Joan de Reus, Reus, Spain; Servicio de Oncología Médica, Complejo Hospitalario Regional y Virgen de la Victoria, Málaga, Spain; Servicio de Oncología Médica, Hospital Universitari i Politècnic la Fe, Valencia, Spain; Servicio de Oncología Médica, Hospital Universitario de Canarias (H.U.C), San Cristóbal De La Laguna, Tenerife Spain; Servicio de Oncología Médica, Hospital General Universitario de Elche y Vega Baja, Elche, Spain; Servicio de Oncología Médica, Hospital Universitario Insular de Gran Canaria, Las Palmas De Gran Canarias, Spain; Servicio de Oncología Médica, Hospital Universitario Puerta de Hierro Majadahonda, Madrid, Spain

**Keywords:** Oncohematology malignancies, Hodgkin lymphoma, Hodgkin lymphoma therapy

## Abstract

Hodgkin lymphoma (HL) is an uncommon B cell lymphoid malignancy representing approximately 10–15 % of all lymphomas. HL is composed of two distinct disease entities; the more commonly diagnosed classical HL and the rare nodular lymphocyte-predominant HL. An accurate assessment of the stage of disease and prognostic factors that identify patients at low or high risk for recurrence are used to optimize therapy. Patients with early stage disease are treated with combined modality strategies using abbreviated courses of combination chemotherapy followed by involved-field radiation therapy, while those with advanced stage disease receive a longer course of chemotherapy often without radiation therapy. High-dose chemotherapy (HDCT) followed by an autologous stem cell transplant (ASCT) is the standard of care for most patients who relapse following initial therapy. Brentuximab vedotin should be considered for patients who fail HDCT with ASCT.

## Methods-methodology

To identify the main topics published in medical literature, a search in "PubMed" and "isiknowledge" (that includes both full papers and abstracts) has been performed. Sentences used were "Hodgkin Lymphoma," "Hodgkin disease" "Hodgkin Lymphoma staging” “Hodgkin Lymphoma treatment," and "Hodgkin Lymphoma new therapies." Main recent reviews on the topics: ESMO clinical guides, NCCN guides, Annual Clinical Updates in Hematology Malignances of the American Journal of Hematology and Italian guideline for HL have been consulted.

## Introduction

Hodgkin Lymphoma (HL, formerly called Hodgkin Disease) is a malignant disease with an incidence of 3.7 (male) and 2.6 (female) cases/100000 (adjusted world estimates rates) in Spain [1]. HL shows an age-related bimodal incidence. The first peak occurs in young adults aged 20–40 years and a much smaller peak occurs after the age of 55 years.

Over the last 4 decades, advances in radiation therapy and the addition of combination chemotherapy have significantly increased the cure rate of patients with HL. Currently, more than 80 % of all newly diagnosed patients younger than 60 years are likely to be cured of their disease. However, most patients with HL die due to acute or late complications, principally treatment induced second solid tumors and cardiovascular disease. This fact must be taken into account when choosing the optimal first-line treatment for an individualized patient.

## Diagnosis

At the time of diagnosis, the majority of patients with HL present with supradiaphragmatic lymphadenopathy. Patients commonly present with cervical, anterior mediastinal, supraclavicular, and axillary lymph node involvement, while the inguinal areas are less frequently involved. Approximately one-third of patients present with systemic symptoms that include fever, night sweats, and weight loss; some patients also present with chronic pruritus. Although the disease most commonly involves contiguous lymph node groups, HL may also affect extranodal tissues by direct invasion or by hematogenous spread. The most commonly involved extranodal sites are the lungs, bone, liver, and bone marrow.

A fine-needle aspirate is inadequate for initial diagnosis. An incisional or excisional biopsy is preferred to provide adequate tissue for different studies (morphology, immunohistochemistry, and molecular biology) but a core-needle biopsy can be considered when excisional biopsy is not possible [2].

HL is a malignancy in that the tumor cells constitute the minority of the cellular population and an inadequate biopsy may fail to include malignant cells in the specimen. To confirm the diagnosis, it is necessary to identify the malignant Reed–Sternberg (RS) cell, which is of follicular center B-cell origin, within the appropriate cellular environment of normal reactive lymphocytes, eosinophils, and histiocytes. Two histological categories have been defined by the WHO classification [3]: the classical variant and the nodular lymphocyte predominant variant.

Classical Hodgkin Lymphoma includes four subtypes: nodular sclerosis, mixed cellularity, lymphocyte-rich and lymphocyte-depleted, and represents about 95 % of all HL cases. Most of cases have expression of CD30 and CD15 but no CD45.

Nodular sclerosis Hodgkin’s lymphoma (NSHL) is the most common subtype of HL and represents about 60 % of cases. Morfologic feature has a partially nodular pattern with fibrous bands separating the nodules in most cases; diffuses areas are common, as is necrosis. The characteristic cell is the lacunar-type RS cell.

Mixed-cellularity Hodgkin’s lymphoma (MCHL) comprises 20–25 % of HL cases. The infiltrate is usually diffuse. RS cells are of the classic type.

Lymphocyte-rich classic Hodgkin’s lymphoma (LRCHL) represents 1 % of HL cases. It has a background infiltrate that consists predominantly of small lymphocytes similar than nodular lymphocyte predominant variant but RS cells are classic or lacunar type.

Lymphocyte-depleted Hodgkin’s lymphoma (LDHL) represents fewer than 1 % of the cases. The infiltrate is diffuse and often hypocellular. It is the most common in individuals positive for human immunodeficiency virus (HIV).

Nodular lymphocyte predominant Hodgkin’s lymphoma is a very rare neoplasm with indolent course and relatively good prognosis. It has a nodular growth pattern and may have diffuse areas. The characteristic neoplastic cell is “pop corn” cell or L&H cell. The background is constituted predominantly by lymphocytes. In contrast to classical HL, the atypical cells are CD45+ and express B-cell-associated antigens (CD20 and CD79a+).

## Staging, prognosis, and response criteria

An accurate assessment of the stage of disease in patients with HL is critical for the selection of the appropriate therapy. The staging system for patients with HL is based on whether the involved lymph nodes are on one or both sides of the diaphragm, the number of involved sites, whether the sites of involvement are bulky, whether there is contiguous extranodal involvement or disseminated extranodal disease, and whether typical systemic symptoms (B symptoms) are present. Fluorodeoxyglucose positron emission tomography (FDGPET) scanning has emerged as an important tool in the staging of patients with HL in that it significantly adds to the staging information obtained using other standard radiographic methods [2].

The Cotswolds modifications of the Ann-Arbor recommendations are the current staging system used for patients with Hodgkin’s lymphoma [4] (Table 1). The recommended staging evaluation should be the following:

**Table 1:** Cotswolds staging classification for Hodgkin’s lymphoma (The Ann Arbor staging system with Cotswolds modifications)

Stage I: Involvement of a single lymph node region (e.g., cervical, axillary, inguinal, mediastinal) or lymphoid structure such as the spleen, thymus, or Waldeyer’s ring
Stage II: Involvement of two or more lymph node regions or lymph node structures on the same side of the diaphragm. Hilar nodes should be considered to be “lateralized” and when involved on both sides, constitute stage II disease. For the purpose of defining the number of anatomic regions, all nodal disease within the mediastinum is considered to be a single lymph node region and hiliar involvement constitutes an additional site of involvement. The number of anatomic regions should be indicated by a subscript (e.g., II-3)
Stage III: Involvement of lymph node regions or lymphoid structures on both sides of the diaphragm. This may be subdivided stage III-1 or III-2: stage III-1 is used for patients with involvement of the spleen or splenic hilar, celiac or portal nodes; and stage III-2 is used for patients with involvement of the paraaortic, iliac, inguinal, or mesenteric nodes
Stage IV: Diffuse or disseminated involvement of one or more extranodal organs or tissue beyond that designated E, with or without associated lymph node involvement
All cases are subclassified to indicate the absence (A) or presence (B) of the systemic symptoms of significant unexplained fever, night sweats, or unexplained weight loss exceeding 10 % of body weight during the 6 months prior to diagnosis
The designation “E” refers to extranodal contiguous extension (i.e., proximal or contiguous extranodal disease) that can be encompassed within an irradiation field appropriate for nodal disease of the same anatomic extent. More extensive extranodal disease is designated stage IV
The subscript “X” is used if bulky disease is present. This is defined as a mediastinal mass with a maximum width that is equal to or greater than one-third of the internal transverse diameter of the thorax at the level of T5/6 interspace or >10 cm maximum dimension of a nodal mass. No subscripts are used in the absence of bulk
Patients can be clinically or pathologically staged. Splenectomy, liver biopsy, lymph node biopsy, and bone marrow biopsy are mandatory for the establishment of pathological stage. The pathologic stage at a given site is denoted by a subscript (e.g., M = bone marrow, H = liver, L = lung, O = bone, P = pleura, and D = skin)

- Clinical evaluation: Age, sex, B Symptoms (fevers to more than 38.3 °C, drenching night sweats or unexplained weight loss more than 10 % of body mass over 6 months), history of malignancy. Fatigue, pruritus, and alcohol-induced pain in patients with HL should also be noted.
- Physical examination includes measurement of accessible nodal groups and the size of the spleen and liver in cm in the midclavicular line.
- Laboratory tests: CBC with differential and platelet count, erythrocyte sedimentation rate (ESR), biochemical tests of liver, bone and renal function, LDH, albumin and calcium concentration. HBV, HCV and HIV tests. Pregnancy test for women of childbearing age.
- Chest X-ray.
- CT scan of the neck, chest, abdomen, and pelvis with contrast.
- PET–CT. In Lugano 2011 the consensus was that PET–CT should be recommended for routine staging as the gold standard [2].
- Bone marrow biopsy from at least one site for patients with clinical stage III–IV or stage II disease with anemia or another blood count depression. However, if PET–CT is performed, a bone marrow biopsy is no longer required for the routine evaluation of patients with HL [2].
- Measures to preserve fertility should be offered to all HL patients before treatment attending to age, patient's wishes and risk of infertility due to therapy.

The predominant factors that determine the initial choice of therapy for HL patients are the histology of the disease (classical HL or nodular lymphocyte-predominant HL), the anatomical stage of disease (limited or advanced disease), and the presence of poor prognostic features.

Among patients with early disease (stage I or II), there is subsequent stratification into favorable and unfavorable prognosis disease based upon the presence or absence of certain clinical features. The two most commonly used definitions of favorable disease are those proposed by the European Organization for the Research and Treatment of Cancer (EORTC) [5] and the German Hodgkin Study Group (GHSG) [6]. The GHSG defines the limited stage favorable prognostic group as patients with no more than two nodal sites; no extranodal extension; no mediastinal mass measuring one-third the maximum thoracic diameter or greater; and ESR less than 50 mm/h (less than 30 mm/h if B symptoms present). Patients with at least one of these factors are considered as unfavorable prognostic early disease. GHSG definition is preferred today because treatment recommendations for these patients are based on the results of GHSG trials.

In contrast, in patients with advanced HL (stage III or IV), disease bulk and other traditional prognostic variables have been found to be less predictive of outcome. A different prognostic scoring system was, therefore, developed for these patients by the International Prognostic Factor Project on advanced HL [7]. This study identified seven variables (age >45 years, presence of stage IV disease, male sex, white blood count >15,000 cells/mL, lymphocyte count <600 cells/mL, albumin <4.0 g/dL, hemoglobin <10.5 g/dL) that predicted patient outcome in a multivariate analysis. Patients with five or more factors were found to have a 5-year freedom from progression of 42 % while patients with no negative prognostic factors had an 84 % likelihood of being free from progression at 5 years.

Response evaluation by contrast-enhanced CT should be carried out after completion of chemotherapy/before RT in early stages and after four cycles of chemotherapy as well as before RT in advanced stages. Final evaluation should be carried out after completion of treatment. Physical examination, laboratory analyses, and contrast-enhanced CT are mandatory. In addition, PET should be carried out at final response evaluation [2]. In patients with early stage, an interim PET made after 2 or 3 cycles of chemotherapy can be useful to avoid RT in selected patients [8] (Table 2).

**Table 2:** Recommendation for the management of classical Hodgkin’s lymphoma

Recommendation 1: A bone marrow biopsy is not required if a PET/CT is performed during routine staging of HL (II, A)
Recommendation 2: Two cycles of ABVD followed by IFRT (20 Gy) is the preferred treatment for favorable early-stage HL (IA). However, for patients with high risk of secondary solid neoplasm, RT could be avoided if a PET CR is achieved after 3–4 ABVD cycles (IB)
Recommendation 3: Four cycles of ABVD followed by IFRT (30 Gy) is the preferred treatment for unfavorable early-stage HL (IA). However, for patients with high risks of secondary solid neoplasm and no bulky disease, RT could be avoided if a PET CR is achieved after 6 ABVD cycles (IIB)
Recommendation 4: Six to eight cycles of ABVD is the preferred treatment for advanced-stage HL (IA). Only patients in PR after chemotherapy should received complementary RT (IA)
Recommendation 5: Salvage chemotherapy followed by high-dose therapy and autologous stem cell transplant is the best option for most patients with relapsing and refractory disease (IB). Brentuximab Vedotin is the preferred option for patients relapsing after ASCT (IIB)
Recommendation 6: Anamnesis and physical examination at 4–6 months intervals for the first 5 years and yearly thereafter is the mainstay of follow-up (IIB). Blood and imaging test are optional and should be individualized (IIB)

***Recommendation 1:****A bone marrow biopsy is not required if a PET/CT is performed during routine staging of HL (II, A).*

## Treatment of classical Hodgkin’s lymphoma

Patients with HL have an excellent outcome with current management approaches. Treatment requires a careful balance between optimum disease control and the risk of long-term treatment-related side effects. Outcome of this population is so successful that even the overall mortality rate from causes other than HL may exceed those seen from Hodgkin’s lymphoma after 10–30 years.

The current standard of care for HL is to have different treatment strategies for HL patients with early stage disease with favorable prognostic features, those with early stage disease but who have poor prognostic features, or those with advanced disease.

### Favorable prognosis early-stage Hodgkin’s lymphoma (Fig. 1)

For decades, extended field radiation therapy (EFRT) has been an essential part of treatment in early-stage HL. However, combined modality treatment (chemotherapy plus less extensive radiotherapy) is the actual standard.

**Fig. 1:**
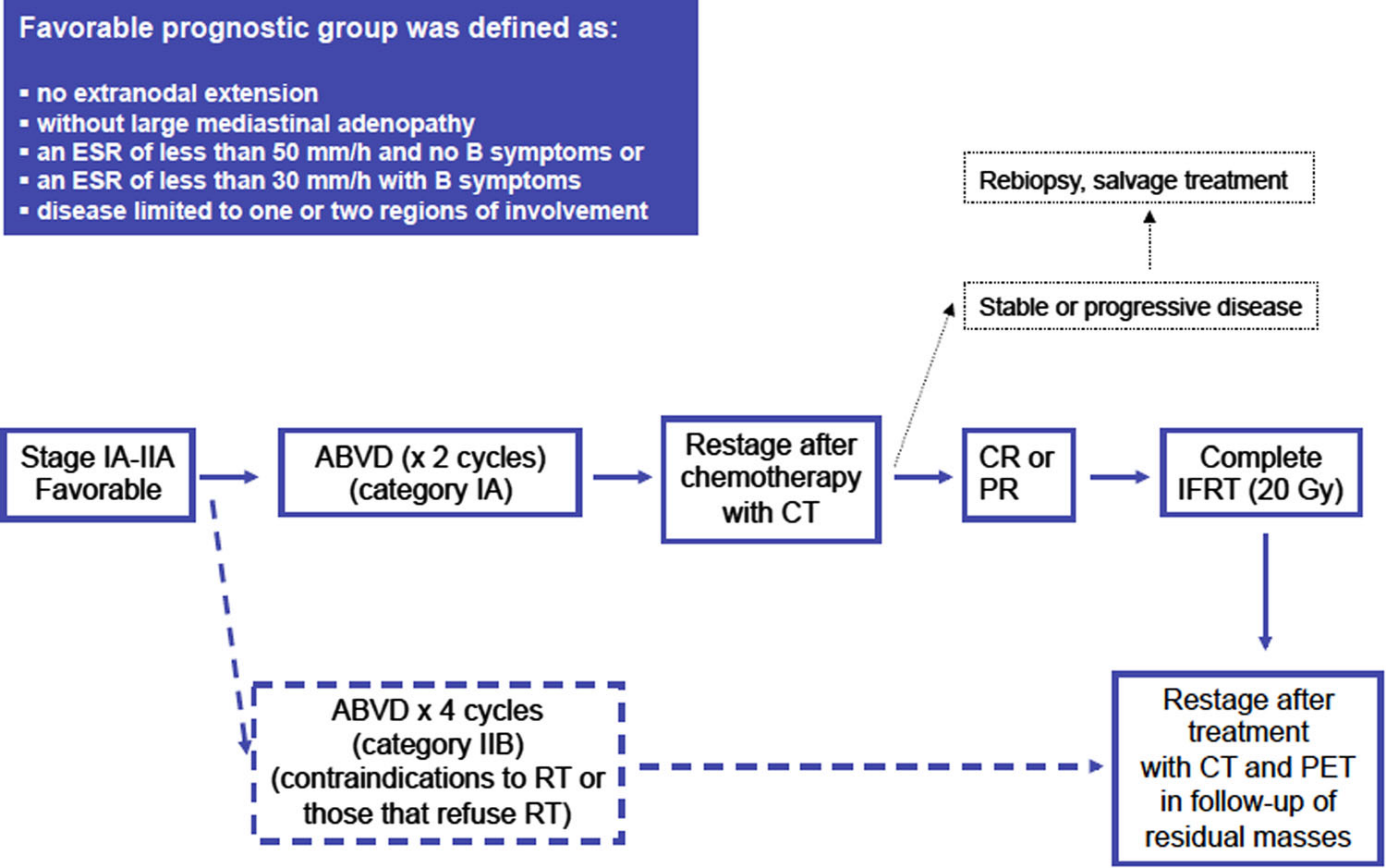
Treatment algorithm for favorable prognosis early (stage I–II) classical Hodgkin’s lymphoma. *Source* Modified from reference [38]

In most of randomized clinical trials, combined modality treatment has resulted in higher rates of freedom from recurrence without differences in overall survival. This lack of overall survival benefit may be related to the effectiveness of salvage chemotherapy after failure of radiation therapy. In a report from the German Hodgkin Study Group (GHSG), HD7 trial, two cycles of ABVD followed by extended-field radiation therapy (EFRT 30 Gy plus 10 Gy to the involved field) was more effective than EFRT alone [6].

Several studies have investigated the reduction of number of cycles of chemotherapy and radiation field size. The EORTC H8F trial compared three cycles of MOPP/ABV hybrid plus involved-field radiation therapy (IFRT) to EFRT. This trial was the first to demonstrate a significant 10-year overall survival benefit in favor of combined modality treatment when compared with radiotherapy alone [9]. In HD10 trial (GHSG), patients were randomized to receive four versus two cycles of ABVD and 30 Gy versus 20 Gy of IFRT. Results after 7.5 years of follow-up showed no differences in survival rates among treatment arms [10].

Chemotherapy alone has also been investigated as a treatment option for patients with early-stage HL. A systematic review of randomized trials showed similar CR rates with a detriment in tumor control and OS in some of them, but this is controversial because both the types of chemotherapy as the volume of radiation therapy utilized was not optimal [11].

Two randomized trials have examined the role of FDG-PET in identifying an early favorable HL patient population in which radiation could be omitted without compromising PFS. Both of these trials used noninferiority designs. The RAPID trial randomized patients who had a negative PET scan after 3 cycles of ABVD to receive additional IFRT or no further therapy. The 3-year PFS rate was superior for the combined treatment arm (93.8 vs. 90.7 %). This study did not demonstrate non-inferiority of the two approaches in PFS [8]. Nevertheless, patients who are PET negative after chemotherapy have a very good outcome with or without consolidation radiotherapy. In EORTC/LYSA/FILH10 trial, involved node radiotherapy (INRT) was omitted in patients with a PET-negative scan after 2 cycles of ABVD. A planned interim analysis for futility led to the closure of the experimental no-radiation arm based on an increased number of progression events when radiation was omitted [12].

***Recommendation 2:****Two cycles of ABVD followed by IFRT (20* *Gy) is the preferred treatment for favorable early*-*stage HL (IA). However, for patients with high risks of secondary solid neoplasm, RT could be avoided if a PET CR is achieved after 3*–*4 ABVD cycles (IB).*

### Unfavorable prognosis early-stage Hodgkin’s lymphoma (Fig. 2)

Different chemotherapeutic regimens have been evaluated in combined modality treatment without identifying any differences in overall survival. ABVD is more effective than MOPP (freedom from progression, FFP) with less hematologic and late gonadal toxicity (H6U trial) but increase in pulmonary toxicity. Less toxic chemotherapy regimens (EVE, EBVP, EBVM) have failed to demonstrate better results.

**Fig. 2:**
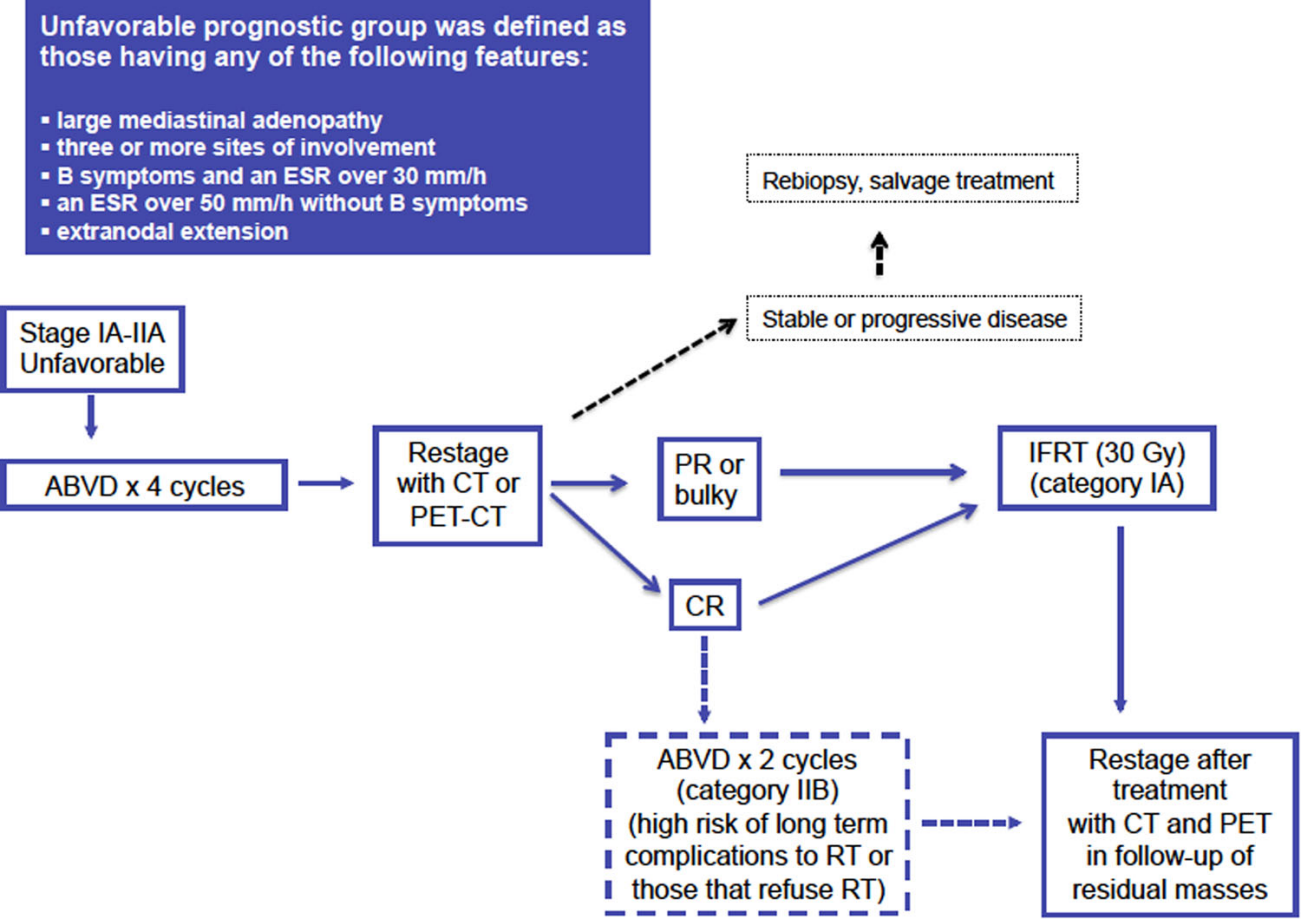
Treatment algorithm for unfavorable prognosis early (stage I–II) classical Hodgkin’s lymphoma. *Source* Modified from reference [38]

To address the issue of the number of cycles of chemotherapy necessary in combined modality treatment, the three-arm EORTC H8U trial compared four versus six cycles of hybrid MOPP/ABV regimen in addition to involved versus extended field radiation without differences in survival [7]. Preliminary results of H9U trial showed similar results in terms of survival between six cycles of ABVD, four cycles of ABVD or four cycles of BEACOPP followed by IFRT 30 Gy in all arms, but increased toxicity was seen with BEACOPP [13].

To determine the radiation dose needs to be applied and looking for an improvement in results with more intensive chemotherapy, the GHSG HD11 trial randomly assigned in a 2 × 2 factorial design to either ABVD or BEACOPPbaseline followed by 20 or 30 Gy of IFRT. BEACOPPbaseline did not significantly improve outcome and four cycles of ABVD should be followed by 30 Gy of IFRT [14].

In HD14 trial, patients were randomly assigned to either four cycles of ABVD or an intensified treatment consisting of two cycles of escalated BEACOPP followed by two cycles of ABVD (2 + 2). Chemotherapy was followed by 30 Gy IFRT in both arms. Intensified chemotherapy achieved a small significant improvement in freedom from treatment failure, mainly in patients with bulky disease, without differences in overall survival. More gonadal and severe acute hematological toxicities were seen with intensive treatment [15].

***Recommendation 3:****Four cycles of ABVD followed by IFRT (30* *Gy) is the preferred treatment for unfavorable early*-*stage HL (IA). However, for patients with high risks of secondary solid neoplasm and no bulky disease, RT could be avoided if a PET CR is achieved after 6 ABVD cycles (IIB).*

### Advanced Hodgkin’s lymphoma (Fig. 3)

Approximately three-quarters of patients with Hodgkin’s lymphoma in advanced stages (stages III and IV) can be cured with chemotherapy. The chemotherapy scheme most widely used is the combination of doxorubicin, bleomicin, vinblastin, and dacarbazine (ABVD). However, results in terms of response and progression free survival are only slightly better than the classic MOPP (meclorethamine, vincristine, procarbazine, and prednisone). The low frequency of long-term toxicities for ABVD, especially second neoplasms and sterility, leads to the abandonment of MOPP and the generalized adoption of ABVD in this setting. Alternating schedules of MOPP and ABVD, and the so-called hybrid scheme (MOPP-ABV) were also compared with ABVD alone, and none of these was associated with a higher overall survival, and the toxicity profile favored ABVD [16].

**Fig. 3:**
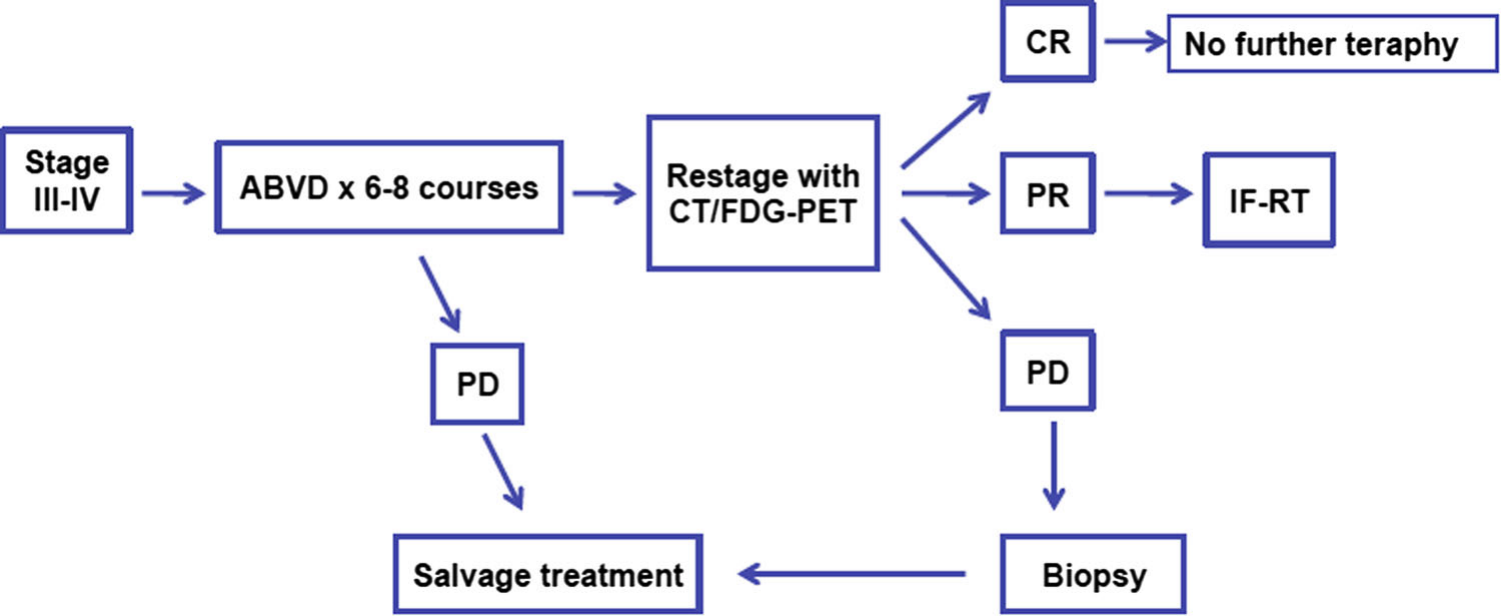
Treatment algorithm for advanced stage disease in classical Hodgkin’s lymphoma. *Source* Modified from reference [38]

The number of cycles of ABVD usually given to treat a patient with advanced Hodgkin’s disease is between six and eight. If there is an early metabolic complete response by Positron Emission Tomography (PET) (e.g., after two or three cycles), six cycles of ABVD are probably sufficient. For slower responders, a total of eight cycles may be needed. However, there is no general agreement on the value of early PET in deciding the total number of chemotherapy cycles to be given [17].

Other chemotherapy combinations have been compared with ABVD with the aim of improving survival in advanced Hodgkin’s lymphoma. Of these, the most relevant are Stanford V and BEACOPP. The Stanford V and BEACOPP original scheme incorporated radiotherapy at the end of chemotherapy in pretreatment-affected areas larger than five cm. Three randomized trials failed to demonstrate the superiority of Stanford V over ABVD. In spite of this, Stanford V was inferior to ABVD when the amount of radiotherapy given was less than that planned in the original phase II trial [18–20]. BEACOPP, and especially its escalated variant (with higher dose of etoposide and cyclophosphamide), demonstrated an improvement in progression free survival, but not in overall survival compared with ABVD, if relapses are treated properly with high-dose chemotherapy. Moreover, BEACOPP was associated with an increased acute and late toxicity (myelotoxicity, secondary leukemia and solid tumors, and sterility) [21].

In patients with advanced stage consolidation radiotherapy can be omitted if a complete response is achieved with chemotherapy [22, 23]. Radiotherapy is recommended if only a partial response is achieved, especially if it is corroborated by PET [23, 24].

***Recommendation 4:****Six to eight cycles of ABVD is the preferred treatment for advanced*-*stage HL (IA). Only patients in PR after chemotherapy should received complementary RT (IA).*

### Therapy of relapsed/resistant disease (Fig. 4)

Approximately 10–15 % of patients in early stage and 20–40 % of patients with advanced stage experience relapse after first-line treatment, generally within the first 12 months. The choice of the best salvage approach should rely on the evaluation of prognostic factors and clinical characteristics of patients. Salvage therapy can achieve durable responses in one-half of these patients.

**Fig. 4:**
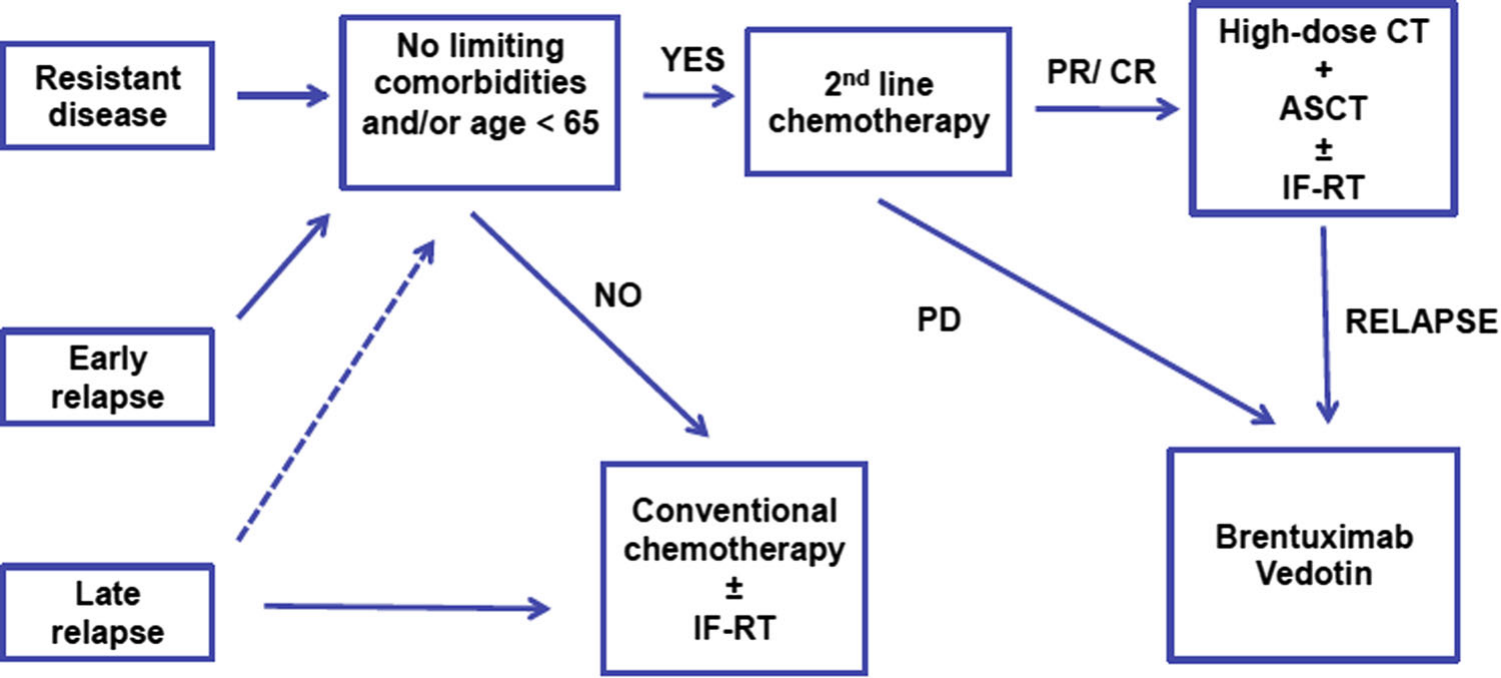
Treatment algorithm for relapsed and resistant disease. *Source* Modified from reference [38]

The length of remission after first-line therapy is the most important prognostic factor and has a significant effect on the success of subsequent salvage treatment. Patients with progressive disease during first line therapy or in the first 3 months after remission are considered to have primary resistant disease and have a cure rate less than 30 %. Early relapse is defined as relapse that occurs within 12 months from remission and late relapse if it occurs beyond this term [25].

At relapse, a new histologic analysis should be performed because of the increased risk of second tumors (NHL or solid tumors) or benign diseases (sarcoidosis and others). Rebiopsy is probably unnecessary in early recurrences with incomplete remissions, especially in symptomatic patients.

Salvage chemotherapy followed by high-dose therapy and autologous stem cell transplant (ASCT) in young patients with relapsing and refractory disease has significantly better results over conventional chemotherapy in terms of disease free survival and is considered standard of care [26–28]. Conventional-dose chemotherapy as salvage treatment is twofold: to achieve a maximum tumor reduction and to mobilize progenitor cells into peripheral blood for subsequent autologous rescue.

Conventional-dose chemotherapy alone has no curative potential in patients with refractory and early-relapsing disease. However, it may be considered the treatment of choice (often followed with radiotherapy) in patients with a late relapse (>12 months after completion of initial therapy), asymptomatic presentation and low burden disease [29]. There are not randomized trials comparing the effectiveness of different conventional salvage chemotherapy regimens and clinical practice varies widely. Regimens most commonly used in this setting are ICE, GDP, GVD, GEM-P, DHAP, ESHAP, and mini-BEAM [29].

With respect to RT, it may have a role when failure occurs in limited nodal sites and prior RT has not been delivered. In addition, RT to residual nodal disease is advisable in patients with residual disease after salvage therapy with or without ASCT. On the contrary, there exists controversy in relation to the eventual benefit of consolidation RT to sites of previous bulky disease [30].

Brentuximab vedotin is an immunotoxin that comprised a CD30 antibody linked to the antitubulin agent monomethyl auristatin E (MMAE). FDA and EMA granted approval to Brentuximab for the treatment of patients with HL after failure of ASCT or after failure of at least two prior multiagent chemotherapy regimens in patients who are not candidates for ASCT. This approval was based on the results of a phase II open-label trial conducted on 102 patients with relapsed or refractory HL after previous ASCT, which were treated with Brentuximab vedotin 1.8 mg/kg every 3 weeks for up to 16 cycles [31]. 75 % of patients achieved an objective response and 34 % of patients achieved CR. After a median follow-up of 33 months, 25 % of the patients with an objective response to brentuximab vedotin (18 out of 103, 16 of them complete responses) were still in remission without the start of new therapy, other than a consolidative allogeneic stem cell transplant (allo-SCT) that was performed in six of 18 patients. The proportion of patients with a best response of CR who remain in remission without a consolidative allo-SCT was 43 % (12/28) [32]. Therefore, consolidative allo-SCT for patients in CR after Brentuximab vedotin should be considered investigational.

Classic Hodgkin’s lymphomas include small numbers of malignant Reed–Sternberg cells within an extensive inflammatory infiltrate, and thus it seems an interesting disease to explore activity of new immunotherapies. In this sense, the genes encoding the PD-1 ligands, *PDL1* and *PDL2,* are key targets of chromosome 9p24.1 amplification, a recurrent genetic abnormality in the nodular sclerosis type of Hodgkin’s lymphoma. JAK-STAT activity induces PD-1 ligand transcription and overexpression of the PD-1 ligands on Reed–Sternberg cells in patients with Hodgkin’s lymphoma. Nivolumab and pembrolizumab are two IgG4 monoclonal antibodies against PD1 that have demonstrated an unexpected activity in two phase 1 studies that included heavily pretreated patients (previous ASCT and Brentuximab in 78 %), with mild toxicities [33, 34]. Other phase 2 trials are ongoing to elucidate clinical effect of these immunotherapies in HL, especially after ASCT.

Allo-SCT is an option in selected patients relapsing after an autologous transplant. Reduced-intensity conditioning (RIC) allo-SCT can induce long-term progression-free survival (PFS), and even curation in a small subset of patients. However, its use is associated with high rates of progression and non-relapse mortality [35].

***Recommendation 5:****Salvage chemotherapy followed by high*-*dose therapy and autologous stem cell transplant is the best option for most patients with relapsing and refractory disease (IB). Brentuximab Vedotin is the preferred option for patients relapsing after ASCT (IIB).*

## Treatment of lymphocyte-predominant Hodgkin’s lymphoma

Lymphocyte-Predominant Hodgkin’s lymphoma (LPHL) is characterized by an indolent course. Usually it involves peripheral lymph nodes with sparing of the mediastinum, retroperitoneum, and the spleen.

Early-stage LPHL has a better prognosis than classical HL. Involved-field radiation therapy (IFRT) 30–36 Gy is recommended for all patients with stage IA or IIA disease. For the rare patients with stage I to II who have B symptoms, combined modality therapy with chemotherapy and IFRT is recommended [37].

Rarely (20 % of cases), patients present as III or IV stage disease, with a concomitantly worse prognosis. Outcome in these cases is similar than classical HL and treatment should be the same.

Late relapses are frequent, regardless of first-line treatment. Biopsy should be performed because high risk of transformation to non-Hodgkin’s lymphoma or classical HL. Limited relapses can receive “involved field” irradiation again.

The monoclonal antibody rituximab has been tested in LPHL with high response rates.

## Follow-up

Follow-up in Hodgkin’s lymphoma is focused on detecting disease relapse and late treatment toxicities. Anamnesis and physical examination at four- to six-month intervals for the first 5 years and yearly thereafter is the mainstay of follow-up. It seems clear that imaging tests in follow-up does not translate into an improvement in survival [36]. However, it is a common practice to perform a CT scan every 6 months for the first 2 years and yearly until the fifth year. A blood test with CBC, ESR, and LDH is usually on a similar schedule to imaging, and some authors recommend performing it yearly for life. If neck radiation therapy is carried out, we suggest including thyroidal function in the blood test. If mediastinal radiation therapy is part of the primary treatment, especially in females younger than twenty, a yearly bilateral breast MRI is recommended from the eighth year post-therapy, in order to screen for breast cancer. The risk of developing lung cancer for heavy smokers who have received mediastinal radiation therapy is well known. Nevertheless it is less clear if a low-dose chest CT scan might be useful in secondary prevention.

***Recommendation 6:****Anamnesis and physical examination at 4*- *to 6*-*month intervals for the first 5 years and yearly thereafter is the mainstay of follow*-*up (IIB). Blood and imaging test are optional and should be individualized (IIB).*

## References

[1] Galcerán J, Ameijide A, Carulla M, Mateos A, Quirós JR, Alemán A, et al. Estimaciones de la incidencia y la supervivencia del cáncer en España y su situación en Europa. Red Española de Registros de Cáncer (REDECAN). 2014.

[2] Cheson BD, Fisher R, Barrington SF, Cavalli F, Schwartz LH, Zucca E (2014). Recommendations for initial evaluation, staging, and response assessment of Hodgkin an Non-Hodgkin Lymphoma: the Lugano Classification. J Clin Oncol.

[3] Swerdlow SH, Campo E, Harris NL, Jaffe ES, Pileri SA, Stein H (2008). World health organization classification of tumours of haematopoietic and lymphoid tissues.

[4] Lister TA, Crowther D, Sutcliffe SB, Glatstein E, Canellos GP, Young RC (1989). Report of a committee convened to discuss the evaluation and staging of patients with Hodgkin’s disease: cotswolds meeting. J Clin Oncol.

[5] Cosset JM, Henry-Amar M, Meerwaldt JH, Carde P, Noordijk EM, Thomas J (1992). The EORTC trials for limited stage Hodgkin’s disease. The EORTC Lymphoma Cooperative Group. Eur J Cancer.

[6] Engert A, Franklin J, Eich HT, Brillant C, Sehlen S, Cartoni C (2007). Two cycles of doxorubicin, bleomycin, vinblastine, and dacarbazine plus extended-field radiotherapy is superior to radiotherapy alone in early favorable Hodgkin’s lymphoma: final results of the GHSG HD7 trial. J Clin Oncol.

[7] Hasenclever D, Diehl V (1998). A prognostic score for advanced Hodgkin’s disease. International Prognostic Factors Project on Advanced Hodgkin’s Disease. N Engl J Med.

[8] Radford J, Illidge T, Counsell N, Hancock B, Pettengell R, Johnson P (2015). Results of a trial of PET-directed therapy for early-stage Hodgkin’s Lymphoma. N Engl J Med.

[9] Fermé C, Eghbali H, Meerwaldt JH, Rieux C, Bosq J, Berger F (2007). Chemotherapy plus involved-field radiation in early-stage Hodgkin’s disease. N Engl J Med.

[10] Engert A, Plütschow A, Eich HT, Lohri A, Dörken B, Borchmann P (2010). Reduced treatment intensity in patients with early-stage Hodgkin’s lymphoma. N Engl J Med.

[11] Straus DJ, Cs Portlock, Qin J, Myers J, Zelenetz AD, Moskowitz C (2004). Results of a prospective randomized clinical trial of doxorubicin, bleomycin, vinblastine, and dacarbazine (ABVD) followed by radiation therapy versus ABVD alone for stages I, II, and IIIA nonbulky Hodgkin disease. Blood.

[12] Raemaekers JMM, André MPE, Federico M, Girinsky T, Oumedaly R, Brusamolino E (2014). Omitting radiotherapy in early positron emission tomography-negative stage I/II Hodgkin lymphoma is associated with an increased risk of early relapse: clinical results of the preplanned interim analysis of the randomized EORTC/LYSA/FIL H10 trial. J Clin Oncol.

[13] Noordijk EM, Thomas J, Fermé C, van ‘t Veer MB, Brice P, Diviné M (2005). First results of the EORTC-GELA H9 randomized trials: the H9-F trial (comparing 3 radiation dose levels) and H9-U trial (comparing 3 chemotherapy schemes) in patients with favorable or unfavorable early stage Hodgkin’s lymphoma (abstract). Proc Am Soc Clin Oncol..

[14] Eich HT, Diehl V, Görgen H, Pabst T, Markova J, Debus J (2010). Intensified chemotherapy and dose-reduced involved-field radiotherapy in patients with early unfavourable Hodgkin’s lymphoma: final analysis of the German Hodgkin Study Group HD11 trial. J Clin Oncol.

[15] von Tresckow B, Plütschow A, Fuchs M, Klimm B, Markova J, Lohri A (2012). Dose-intensification in early unfavorable Hodgkin’s lymphoma: final analysis of the German Hodgkin Study Group HD14 trial. J Clin Oncol.

[16] Canellos GP, Anderson JR, Propert KJ, Nissen N, Cooper MR, Henderson ES (1992). Chemotherapy of advanced Hodgkin’s disease with MOPP, ABVD or MOPP alternating with ABVD. N Engl J Med.

[17] Hutchings M, Kostakoglu L, Zaucha JM, Malkowski B, Biggi A, Danielewicz I (2014). In vivo treatment sensitivity testing with positron emission tomography/computed tomography after one cycle of chemotherapy for Hodgkin lymphoma. J Clin Oncol.

[18] Hoskin PJ, Lowry L, Horwich A, Jack A, Mead B, Hancock BW (2009). Randomized comparison of the stanford v regimen and ABVD in the treatment of advanced Hodgkin’s lymphoma: United Kingdom National Cancer Research Institute Lymphoma Group Study ISRCTN 64141244. J Clin Oncol.

[19] Chisesi T, Bellei M, Luminari S, Montanini A, Marcheselli L, Levis A (2011). Long-term follow-up analysis of HD9601 trial comparing ABVD versus Stanford V versus MOPP/EBV/CAD in patients with newly diagnosed advanced-stage Hodgkin’s lymphoma: a study from the Intergruppo Italiano Linfomi. J Clin Oncol.

[20] Gordon LI, Hong F, Fisher RI, Bartlett NL, Connors JM, Gascoyne RD (2013). Randomized phase III trial of ABVD versus Stanford V with or without radiation therapy in locally extensive and advanced-stage Hodgkin lymphoma: an Intergroup Study Coordinated by the Eastern Cooperative Oncology Group (E2496). J Clin Oncol.

[21] Bauer K, Skoetz N, Monsef I, Engert A, Brillant C (2011). Comparison of chemotherapy including escalated BEACOPP versus chemotherapy including ABVD for patients with early unfavourable or advanced stage Hodgkin lymphoma. Cochrane Database Syst Rev.

[22] Loeffler M, Brosteanu O, Hasenclever D, Sextro M, Assouline D, Bartolucci AA (1998). Meta-analysis of chemotherapy versus combined modality treatment trials in Hodgkin’s disease. International Database on Hodgkin’s Disease Overview Study Group. J Clin Oncol.

[23] Engert A, Haverkamp H, Kobe C, Markova J, Renner C, Ho A (2012). Reduced-intensity chemotherapy and PET-guided radiotherapy in patients with advanced stage Hodgkin’s lymphoma (HD15 trial): a randomised, open-label, phase 3 non-inferiority trial. Lancet.

[24] Aleman BMP, Raemaekers JMM, Tirelli U, Bortolus R, van ‘tVeer MB, Lybeert ML (2003). Involved-field radiotherapy for advanced Hodgkin’s lymphoma. N Engl J Med.

[25] Josting A, Franklin J, May M, Koch P, Beykirch MK, Heinz J (2002). New prognostic score based on treatment outcome of patients with relapsed Hodgkin’s lymphoma registered in the database of the German Hodgkin’s lymphoma study group. J Clin Oncol.

[26] Linch DC, Winfield D, Goldstone AH, Moir D, Hancock B, McMillan A (1993). Dose intensification with autologous bone marrow transplantation in relapsed and resistant Hodgkin’s disease: results of a BLNI randomized trial. Lancet.

[27] Schmitz N, Pfistner B, Sextro M, Sieber M, Carella AM, Haenel M (2002). Aggressive conventional chemotherapy compared with high-dose chemotherapy with autologous haemopoietic stem-cell transplantation for relapsed chemosensitive Hodgkin’s disease: a randomized trial. Lancet.

[28] Rancea M, Monsef I, von Tresckow M, Engert A, Skoetz N (2013). High-dose chemotherapy followed by autologous stem cell transplantation for patients with relapsed/refractory Hodgkin lymphoma. Cochrane Database Syst Rev.

[29] Collins GP, Parker AN, Pocock C, Kayani I, Sureda A, Illidge T (2014). Guideline on the management of primary resistant and relapsed classical Hodgkin lymphoma. Br J Haematol.

[30] Biswas T, Culakova E, Friedberg JW, Kelly JL, Dhakal S, Liesveld J (2012). Involved field radiation therapy following high dose chemotherapy and autologous stem cell transplant benefits local control and survival in refractory or recurrent Hodgkin lymphoma. Radiother Oncol.

[31] Pro B, Advani R, Brice P, Bartlett NL, Rosenblatt JD, IIIidge T (2012). Brentuximab vedotin (SGN-35) in patients with relapsed or refractory systemic anaplastic large-cell lymphoma: results of a phase II study. J Clin Oncol.

[32] Gopal AK, Chen R, Smith SE, Ansell SM, Rosenblatt JD, Savage KJ (2015). Durable remissions in a pivotal phase 2 study of brentuximab vedotin in relapsed or refractory Hodgkin lymphoma. Blood.

[33] Ansell SM, Lesokhin AM, Borrello I, Halwani A, Scott EC, Gutierrez M (2015). PD-1 blockade with nivolumab in relapsed or refractory Hodgkin’s lymphoma. N Engl J Med.

[34] Ansell SM (2015). Targeting immune checkpoints in lymphoma. Curr Opin Hematol.

[35] Sureda A, Canals C, Arranz R, Caballero D, Ribera JM, Brune M (2012). Allogeneic stem cell transplantation after reduced intensity conditioning in patients with relapsed or refractory Hodgkin’s lymphoma. Results of the HDR-ALLO study—a prospective clinical trial by the Grupo Español de Linfomas/Trasplante de Medula Osea (GEL/TAMO) and the Lymphoma Working Party of the European Group for Blood and Marrow Transplantation. Haematologica.

[36] Gandikota N, Hartridge-Lambert S, Migliacci JC, Yahalom J, Portlock CS, Schöder H (2015). Very low utility of surveillance imaging in early-stage classic Hodgkin lymphoma treated with a combination of doxorubicin, bleomycin, vinblastine, and dacarbazine and radiation therapy. Cancer.

[37] Advani RH, Hoppe RT (2015). Management of nodular lymphocyte predominant Hodgkin lymphoma. Hematol Oncol.

[38] Quero Blanco C, García Arroyo R, Provencio Pulla M, Rueda Domínguez A, Isla Casado D (2010). SEOM clinical guidelines for the treatment of Hodgkin’s lymphoma. Clin Transl Oncol.

